# Preference of Women for Gestational Diabetes Screening Method According to Tolerance of Tests and Population Characteristics

**DOI:** 10.3389/fendo.2021.781384

**Published:** 2021-11-08

**Authors:** Lore Raets, Marie Vandewinkel, Paul Van Crombrugge, Carolien Moyson, Johan Verhaeghe, Sofie Vandeginste, Hilde Verlaenen, Chris Vercammen, Toon Maes, Els Dufraimont, Nele Roggen, Christophe De Block, Yves Jacquemyn, Farah Mekahli, Katrien De Clippel, Annick Van Den Bruel, Anne Loccufier, Annouschka Laenen, Roland Devlieger, Chantal Mathieu, Katrien Benhalima

**Affiliations:** ^1^ Department of Endocrinology, University Hospital Gasthuisberg, KU Leuven, Leuven, Belgium; ^2^ Medicine, KU Leuven, Leuven, Belgium; ^3^ Department of Endocrinology, OLV ziekenhuis Aalst-Asse-Ninove, Aalst, Belgium; ^4^ Department of Obstetrics & Gynecology, UZ Gasthuisberg, KU Leuven, Leuven, Belgium; ^5^ Department of Obstetrics & Gynecology, OLV ziekenhuis Aalst-Asse-Ninove, Aalst, Belgium; ^6^ Department of Endocrinology, Imelda ziekenhuis, Bonheiden, Belgium; ^7^ Department of Obstetrics & Gynecology, Imelda ziekenhuis, Bonheiden, Belgium; ^8^ Department of Endocrinology-Diabetology-Metabolism, Antwerp University Hospital, Edegem, Belgium; ^9^ Department of Obstetrics & Gynecology, Antwerp University Hospital, Edegem, Belgium; ^10^ Department of Endocrinology, Kliniek St-Jan Brussel, Brussel, Belgium; ^11^ Department of Obstetrics & Gynecology, Kliniek St-Jan Brussel, Brussel, Belgium; ^12^ Department of Endocrinology, AZ St Jan Brugge, Brugge, Belgium; ^13^ Department of Obstetrics & Gynecology, AZ St Jan Brugge, Brugge, Belgium; ^14^ Center of Biostatics and Statistical bioinformatics, KU Leuven, Leuven, Belgium

**Keywords:** gestational diabetes mellitus, preference for screening method, tolerance, glucose challenge test, two-step screening, one-step screening, oral glucose tolerance test

## Abstract

**Aims:**

To determine the preferred method of screening for gestational diabetes mellitus (GDM).

**Methods:**

1804 women from a prospective study (NCT02036619) received a glucose challenge test (GCT) and 75g oral glucose tolerance test (OGTT) between 24-28 weeks. Tolerance of screening tests and preference for screening strategy (two-step screening strategy with GCT compared to one-step screening strategy with OGTT) were evaluated by a self-designed questionnaire at the time of the GCT and OGTT.

**Results:**

Compared to women who preferred one-step screening [26.2% (472)], women who preferred two-step screening [46.3% (834)] were less often from a minor ethnic background [6.0% (50) *vs*. 10.7% (50), p=0.003], had less often a previous history of GDM [7.3% (29) *vs*. 13.8% (32), p=0.008], were less often overweight or obese [respectively 23.1% (50) *vs*. 24.8% (116), p<0.001 and 7.9% (66) *vs*. 18.2% (85), p<0.001], were less insulin resistant in early pregnancy (HOMA-IR 8.9 (6.4-12.3) *vs*. 9.9 (7.2-14.2), p<0.001], and pregnancy outcomes were similar except for fewer labor inductions and emergency cesarean sections [respectively 26.6% (198) *vs*. 32.5% (137), p=0.031 and 8.2% (68) *vs*. 13.0% (61), p=0.005]. Women who preferred two-step screening had more often complaints of the OGTT compared to women who preferred one-step screening [50.4% (420) *vs*. 40.3% (190), p<0.001].

**Conclusions:**

A two-step GDM screening involving a GCT and subsequent OGTT is the preferred GDM screening strategy. Women with a more adverse metabolic profile preferred one-step screening with OGTT while women preferring two-step screening had a better metabolic profile and more discomfort of the OGTT. The preference for the GDM screening method is in line with the recommended Flemish modified two-step screening method, in which women at higher risk for GDM are recommended a one-step screening strategy with an OGTT, while women without these risk factors, are offered a two-step screening strategy with GCT.

**Clinical Trial Registration:**

NCT02036619 https://clinicaltrials.gov/ct2/show/NCT02036619

## Introduction

Gestational diabetes mellitus (GDM) is defined as diabetes diagnosed in the second or third trimester of pregnancy, given that overt diabetes early in pregnancy has been excluded (1). Adverse pregnancy outcomes, such as large-for-gestational age (LGA) infants, preeclampsia and cesarean sections, can be reduced by treatment of GDM between 24-28 weeks of pregnancy (2, 3). Women with a history of GDM have a seven-fold increased risk of developing type 2 diabetes mellitus (T2DM) compared to normal glucose tolerant women (NGT). These women also have a higher risk of developing a metabolic syndrome and cardiovascular events later in life compared to NGT women (4–7).

A universal one-step approach for GDM screening is recommended by the ‘International Association of Diabetes and Pregnancy Study Groups’ (IADPSG) with the 75g oral glucose tolerance test (OGTT) between 24-28 weeks of pregnancy (8). The World Health Organization (WHO) adopted these recommendations in 2013, whereby the IADPSG guidelines are now commonly referred to as the 2013 WHO criteria (9). However, using this one-step approach leads to an increased workload and possible unnecessary medicalization of care. Therefore, many European countries still use selective screening for GDM based on risk factors or recommend a two-step screening strategy with a glucose challenge test (GCT) (10). In addition, evidence is lacking that treatment of GDM based on the IADPSG screening strategy improves pregnancy outcomes compared to other screening strategies (11, 12). Moreover, a GCT is generally better tolerated than an OGTT and has the advantage that it can be performed in a non-fasting state (12). A modified two-step screening strategy with GCT combined with risk-factors was proposed based on the BEDIP-N study in Flanders. This allows women at higher risk for GDM (women with a history of GDM, obesity and/or impaired fasting glycaemia in early pregnancy) to receive a one-step screening strategy with an OGTT, while women without these risk factors, are offered a two-step screening strategy with GCT (13). However, data are lacking on which GDM screening method and which screening test pregnant women prefer. We aimed therefore to determine which GDM screening method (a two-step screening strategy with a GCT or a one-step screening approach with a 75g OGTT) participants preferred. In addition, we specifically aimed to determine the preference of GDM screening method according to the tolerance for the different screening tests and in relation to the population characteristics.

## Subjects and Methods

### Study Design and Setting

This article is a sub-analysis of the BEDIP-N cohort (NCT02036619). The BEDIP-N study was a multicentric prospective cohort study that has previously been described in detail (10, 14–17). The BEDIP-N study was approved by the Institutional Review Boards of all participating centers and all investigations have been carried out in accordance with the principles of the Declaration of Helsinki as revised in 2008. Before inclusion to the study, participants provided informed consent. Participants were enrolled between 6 and 14 weeks of pregnancy, when fasting plasma glucose (FPG) was measured. Women with impaired fasting glucose or diabetes in early pregnancy according to the American Diabetes Association (ADA) criteria, were excluded. Women without (pre)diabetes received universal screening for GDM between 24-28 weeks of pregnancy with both a non-fasting 50g GCT and a 75g 2-hour OGTT. Results of the GCT were blinded for participants and health care provides, so all participants received an OGTT irrespective of the GCT result. The diagnosis of GDM was based on the IADPSG/2013 WHO criteria (9, 14, 15). For treatment of GDM, the ADA-recommended glycemic targets were used (9). If targets were not reached within two weeks after the start of lifestyle measures, insulin was started. Women with GDM received an invitation for a postpartum 75g OGTT 6 to 16 weeks after delivery. The ADA criteria were used to define diabetes and glucose intolerance [impaired fasting glycemia (IFG) and/or impaired glucose tolerance (IGT)] (9, 14).

### Study Visits and Measurements

Baseline characteristics and obstetrical history were collected at first visit (14). At first visit and at the time of the OGTT, anthropometric measurements were obtained and several self-administered questionnaires were completed (14). The tolerance for the GCT and OGTT was evaluated by a self-designed questionnaire evaluating any discomfort or complaint with the test such as bad taste, nausea, vomiting, dizziness or abdominal pain. In addition, at the time of the OGTT the questionnaire also evaluated whether women considered it cumbersome to have to be fasting for the test, which screening test they would prefer (non-fasting GCT or the fasting OGTT) and whether they preferred a two-step screening strategy with GCT or a one-step screening approach with 75g OGTT. The CES-D questionnaire to evaluate symptoms of depression was completed at time of the OGTT (before the diagnosis of GDM was communicated), and at the postpartum OGTT for women with GDM (with a score ≥16 being suggestive for clinical depression (18). At first visit and at the time of the OGTT, a food questionnaire was used to question servings per week of different important food categories and beverages (19). Less healthy consumption was assigned 0 or -1 points. By summing up the points for all 14 food groups, the diet score could range from -12 to 15. At the time of the OGTT, the International Physical Activity Questionnaire (IPAQ) questionnaire (validated for the Belgian population) assessed physical activity (14, 20). Results of the IPAQ were reported in categories (low, moderate or high activity levels) as previously reported (21). In early postpartum, participants completed the SF-36 health survey (22)

Blood pressure (BP) was measured twice, with 5 minutes between each measurement using an automatic BP monitor. A BMI ≥ 25 kg/m² was defined as overweight, a BMI ≥ 30 kg/m² was defined as obese based on the BMI at first prenatal visit. During the first perinatal visit, a fasting blood test was taken to measure FPG, insulin, lipid profile (total cholesterol, HDL and LDL cholesterol, triglycerides) and HbA1c. The homeostasis model assessment of insulin resistance (HOMA-IR) and beta-cell function (HOMA-B) was measured in early pregnancy (23). During the OGTT, glucose and insulin were measured fasting, at 30min, 60min and 120min. The results of glucose and insulin levels during the OGTT were used to calculate the Matsuda index, which is a measure of whole body insulin sensitivity (24). Furthermore, a fasting lipid profile, HbA1c and different indices of beta-cell function [HOMA-B, the insulinogenic index divided by HOMA-IR and the insulin secretion-sensitivity index-2 (ISSI-2)] were also measured at time of the OGTT (14, 23, 25–27).

### Pregnancy and Delivery Outcome Data

The following pregnancy outcome data were collected: gestational age, preeclampsia (*de novo* BP ≥140/90mmHg > 20 weeks with proteinuria or signs of end-organ dysfunction), gestational hypertension (*de novo* BP ≥140/90mmHg > 20 weeks), type of labor and type of delivery, birth weight, macrosomia (>4 kg), birth weight ≥4.5 kg, LGA defined as birth weight >90 percentile according to standardized Flemish birth charts adjusted for sex of the baby and parity (28), small-for-gestational age (SGA) defined as birth weight <10 percentile according to standardized Flemish birth charts adjusted for sex of the baby and parity (28), preterm delivery (<37 completed weeks), 10min Apgar score, shoulder dystocia, neonatal respiratory distress syndrome, neonatal jaundice, congenital anomalies and admission on the neonatal intensive care unit (NICU) (14). Irrespective of the need for intravenous administration of glucose and admission on the NICU, a glycemic value <2.2mmol/l was considered as a neonatal hypoglycemia across all centers. Admission to the NICU was decided by the neonatologist in line with normal routine in each center. The difference in weight between first prenatal visit and the time of the OGTT was calculated as early weight gain. The total gestational weight gain was calculated as the difference in weight between first prenatal visit and the delivery. Excessive total gestational weight gain was defined according to the 2009 Institute of Medicine (IOM) guidelines (29).

### Statistical Analysis

Descriptive statistics were presented as frequencies and percentages for categorical variables and means with standard deviations or medians with interquartile range for continuous variables. Categorical variables were analyzed using the Chi-square test or the Fisher exact test in case of low (<5) cell frequencies, whereas continuous variables were analyzed using the Kruskal-Wallis test for not normally distributed variables or One-way ANOVA test for normally distributed variables. A p-value <0.05 was considered significant. Analyzes were performed by statistician A. Laenen by using SAS software.

## Results

### Preference for the GDM Screening Method

1803 women received both a GCT and an OGTT in the BEDIP-N study. Of all women, 46.3% (834) preferred two-step screening with a GCT, 26.2% (472) preferred a one-step screening strategy with an OGTT and 27.6% (497) had no clear preference. The most preferred screening test was a GCT, (by 54.8% (989) of all participants), while only 6.2% (112) preferred an OGTT and 39% (703) had no clear preference.

### Tolerance of Screening Tests

Women who preferred a two-step screening strategy tolerated the GCT in general significantly better than the OGTT compared to women who preferred a one-step screening approach and compared to women without clear preference (**Table 1**). In addition, women who preferred a two-step screening indicated that it was more cumbersome to be fasting for the OGTT compared to women who preferred a one-step screening strategy or had no clear preference and they reported more complaints of the OGTT (**Table 1**). The most common complaint during an OGTT was nausea (in each group more than half of all women reported nausea). There were no significant differences in the type of complaints for the OGTT between both groups, except that more women who preferred one-step screening reported abdominal pain compared to women who preferred two-step screening [8.4% (16) *vs*. 3.1% (13), p=0.004] (**Table 1**).

**Table 1:** Comparison of tolerance for screening tests between women who prefer two-step screening compared to women who prefer one-step screening with OGTT or without clear preference.

	Preference two-step screening	Preference one-step OGTT	No preference	Pairwise comparisons		
				1 *vs*2	1 *vs* 3	2 *vs* 3
	N = 834 (46.3%)	N = 472 (26.2%)	N = 497 (27.6%)			
% Any discomfort of GCT:				**0.003**	0.290	0.086
No	82.0 (667)	75.0 (342)	79.7 (388)			
Yes	18.0 (146)	25.0 (114)	20.3 (99)			
% Bad taste				0.621	0.494	0.844
No	74.7 (109)	71.9 (82)	70.7 (70)			
Yes	25.3 (37)	28.1 (32)	29.3 (29)			
% Nausea				0.626	0.168	0.080
No	54.8 (80)	51.7 (59)	63.6 (63)			
Yes	45.2 (66)	48.2 (55)	36.4 (36)			
% Dizziness or feeling faint				0.916	0.870	0.802
No	61.6 (90)	62.3 (71)	60.6 (60)			
Yes	38.4 (56)	37.7 (43)	39.4 (39)			
% Vomiting				0.278	0.721	0.480
No	97.3 (142)	99.1 (113)	98.0 (97)			
Yes	2.7 (4)	0.9 (1)	2.0 (2)			
% Abdominal pain GCT				0.759	0.628	0.861
No	97.9 (143)	97.4 (111)	97.0 (96)			
Yes	2.0 (3)	2.6 (3)	3.0 (3)			
% Any discomfort of OGTT:				**<.001**	**<.001**	0.084
No	49.6 (413)	59.7 (281)	65.0 (322)			
Yes	50.4 (420)	40.3 (190)	34.9 (173)			
% Bad taste				0.196	0.121	**0.016**
No	70.0 (294)	64.7 (123)	76.3 (132)			
Yes	30.0 (126)	35.3 (67)	23.7 (41)			
% Nausea				0.746	0.301	0.536
No	43.3 (182)	44.7 (85)	48.0 (83)			
Yes	56.7 (238)	55.3 (105)	52.0 (90)			
% Dizziness or feeling faint				0.260	0.463	0.759
No	49.3 (207)	54.2 (103)	52.6 (91)			
Yes	50.7 (213)	45.8 (87)	47.4 (82)			
% Vomiting				0.802	0.855	0.960
No	95.7 (402)	95.3 (181)	95.4 (165)			
Yes	4.3 (18)	4.7 (9)	4.6 (8)			
% Abdominal pain				**0.004**	0.067	0.454
No	96.9 (407)	91.6 (174)	93.6 (162)			
Yes	3.1 (13)	8.4 (16)	6.4 (11)			
% Cumbersome to be fasting				**<.001**	**<.001**	**<.001**
No	43.0 (358)	64.3 (303)	78.4 (388)			
Yes	57.0 (474)	35.7 (168)	21.6 (107)			

OGTT, oral glucose tolerance test; GCT, glucose challenge test; Categorical variables are presented as frequencies %(n); Differences are considered significant at p-value < 0.05. Bold value means that this is significant, meaning that the p-value < 0.05.

Women who preferred a GCT test had less complaints of the GCT compared to women with a preference for the OGTT [18.6% (180) *vs*. 33.9% (37), p<0.001]. Significantly more women who preferred a GCT found it cumbersome to be fasting for the OGTT compared to women who preferred an OGTT or had no clear preference [respectively 58.7% (579) *vs*. 34.2% (38), p<0.001 and 58.7% (579) *vs*. 18.7% (131), p<0.001] (**Table 2**).

**Table 2:** Comparison of tolerance for screening tests between women who prefer a GCT compared to women who prefer an OGTT or without clear preference.

	Preference OGTT	Preference GCT	No preference	Pairwise comparisons		
	N = 112 (6.21%)	N = 989 (54.82%)	N = 703 (38.97%)	1 *vs* 2	1 *vs* 3	2 *vs* 3
% Any discomfort of GCT:				**<.001**	**0.002**	0.259
No	66.1 (72)	81.4 (787)	79.1 (539)			
Yes	33.9 (37)	18.6 (180)	20.8 (142)			
% Bad taste				0.102	**0.002**	**0.004**
No	89.2 (33)	77.2 (139)	62.7 (89)			
Yes	10.8 (4)	22.8 (41)	37.3 (53)			
% Nausea				0.233	0.172	0.748
No	45.9 (17)	56.7 (102)	58.4 (83)			
Yes	54.0 (20)	43.3 (78)	41.5 (59)			
% Dizziness or feeling faint				0.666	0.706	0.190
No	62.2 (23)	58.3 (105)	65.5 (93)			
Yes	37.8 (14)	41.7 (75)	34.5 (49)			
% Vomiting				0.167	0.143	0.852
No	94.6 (35)	98.3 (177)	98.6 (140)			
Yes	5.4 (2)	1.7 (3)	1.4 (2)			
% Abdominal pain				0.282	0.279	0.947
No	94.6 (35)	97.8 (176)	97.9 (139)			
Yes	5.4 (2)	2.2 (4)	2.1 (3)			
% Any discomfort of OGTT:				0.872	**0.002**	**<.001**
No	50.9 (56)	50.1 (495)	66.5 (466)			
Yes	49.1 (54)	49.9 (493)	33.5 (235)			
% Bad taste				0.949	0.790	0.530
No	70.4 (38)	70.8 (349)	68.5 (161)			
Yes	29.6 (16)	29.2 (144)	31.5 (74)			
% Nausea				0.562	0.165	0.107
No	38.9 (21)	43.0 (212)	49.4 (116)			
Yes	61.1 (33)	57.0 (281)	50.6 (119)			
% Vomit OGTT test				0.334	0.184	0.502
No	92.6 (50)	95.5 (471)	96.6 (227)			
Yes	7.4 (4)	4.5 (22)	3.4 (8)			
% Dizziness or feeling faint				0.382	0.840	0.230
No	55.6 (30)	49.3 (243)	54.0 (127)			
Yes	44.4 (24)	50.7 (250)	46.0 (108)			
% Abdominal pain				**0.001**	0.132	**0.042**
No	87.0 (47)	96.5 (476)	93.2 (219)			
Yes	13.0 (7)	3.4 (17)	6.8 (16)			
% Cumbersome to be fasting				**<.001**	**<.001**	**<.001**
No	67.8 (73)	41.3 (408)	81.3 (570)			
Yes	34.2 (38)	58.7 (579)	18.7 (131)			

OGTT, oral glucose tolerance test; GCT, glucose challenge test; Categorical variables are presented as frequencies %(n); Differences are considered significant at p-value<0.05. Bold value means that this is significant, meaning that the p-value < 0.05.

### Characteristics of Women According to the Preference of GDM Screening Method

Compared to women who preferred one-step screening, women who preferred two-step screening, had less often a minor ethnic background, had less often a low income, had less often a first degree family history of GDM or a previous history of GDM [7.3% (29) *vs*. 13.8% (32), p=0.008], had a lower BMI [23.9 ± 4.0 *vs*. 25.4 ± 5.3, p<0.001), were less often overweight or obese [respectively 23.1% (50) *vs*. 24.8% (116), p<0.001 and 7.9% (66) *vs*. 18.2% (85), p<0.001], and were less insulin resistant in early pregnancy (HOMA-IR 8.9 (6.4-12.3) *vs*. 9.9 (7.2-14.2), p<0.001] (**Table 3**). There was no difference in the multiparity rate between both groups.

**Table 3:** Comparison of characteristics and pregnancy outcomes between women who prefer two-step screening compared to women who prefer one-step screening with OGTT or without clear preference.

	Preference two-step screening	Preference one-step OGTT	No preference	Pairwise comparisons		
	N = 834 (46.3%)	N = 472 (26.2%)	N = 497 (27.6%)			
				1 *vs* 2	1 *vs* 3	2 *vs* 3
**General**						
Age (years)	30.6 ± 3.8	30.8 ± 4.0	31.0 ± 4.4	0.180	0.071	0.717
% Minor ethnicities	6.0 (50)	10.7 (50)	12 (59)	**0.003**	**<.001**	0.523
% multiparity	47.4 (395)	47.9 (226)	46.7 (232)	0.857	0.809	0.708
% Highest education:				0.105	**<.001**	**0.002**
primary school	0.4 (3)	1.1 (5)	2.7 (13)			
till 15 years	2.5 (21)	3.9 (18)	7.6 (37)			
high school	14.1 (117)	17.5 (80)	22.4 (109)			
bachelor	43.5 (360)	41.0 (188)	39.2 (191)			
master	39.5 (327)	36.5 (167)	28.1 (137)			
% paid job	94.3 (784)	91.7 (431)	88.4 (436)	0.065	**<.001**	0.091
% low monthly net income family <1500 euro	1.8 (15)	5.0 (23)	6.6 (32)	**0.003**	**<.001**	0.276
% 1500-5000 euro	90.8 (739)	89.6 (415)	89.7 (435)			
% >5000 euro	7.4 (60)	5.4 (25)	3.7 (18)			
Low income (<1500 euro)				**0.003**	**<.001**	0.331
% No	98.2 (799)	95.0 (440)	93.4 (453)			
% Yes	1.8 (15)	5.0 (23)	6.6 (32)			
%living without partner	15.0 (124)	22.5 (106)	17.8 (88)	**<.001**	0.176	0.064
% smoking before pregnancy	29.1 (242)	31.3 (147)	28.2 (139)	0.415	0.719	0.295
% smoking during pregnancy	3.7 (31)	3.0 (14)	3.8 (19)	0.474	0.917	0.459
% First degree family history of diabetes	12.0 (97)	13.0 (60)	13.2 (64)	0.588	0.502	0.915
% First degree family history of GDM	3.8 (29)	6.4 (28)	3.4 (16)	**0.039**	0.773	**0.041**
% History of GDM*	7.3 (29)	13.8 (32)	5.5 (13)	**0.008**	0.392	**0.002**
%History of impaired glucose intolerance	1.8 (13)	1.0 (4)	1.1 (5)	0.267	0.366	0.820
**6-14 weeks visit**						
Week first visit with FPG	11.8 ± 1.8	11.9 ± 1.7	12.0 ± 1.8	**0.027**	**0.002**	0.454
BMI (Kg/m²)	23.9 ± 4.0	25.4 ± 5.3	25.4 ± 4.7	**<.001**	**<.001**	0.426
% Overweight	23.1 (50)	24.8 (116)	29.3 (145)	**<.001**	**<.001**	0.430
% Obesity	7.9 (66)	18.2 (85)	15.3 (76)			
Waist circumference (cm)	85.5 ± 10.0	88.5 ± 12.6	88.4 ± 11.6	**<.001**	**<.001**	0.943
% Waist ≥80cm	71.9 (586)	76.4 (346)	78.1 (367)	**<.001**	**0.003**	0.627
Weight gain (first visit till OGTT) (Kg)	7.2 ± 2.9	6.9 ± 3.6	7.1 ± 3.6	0.534	0.796	0.424
Systolic blood pressure (mmHg)	114.6 ± 10.3	115.7 ± 10.8	115.1 ± 11.0	0.093	0.377	0.483
Diastolic blood pressure (mmHg)	70.4 ± 7.8	70.8 ± .8.3	70.5 ± 8.6	0.401	0.949	0.463
Total Score lifestyle						
Physical activity	1.0 (0.0-2.0)	1.0 (0.0-2.0)	1.0 (0.0-2.0)	0.470	0.432	0.190
Diet	2.0 (0.0-4.0)	2.0 (0.0-5.0)	2.0 (0.0-4.0)	0.658	0.347	0.240
Fasting glycaemia (mg/dl)	82.0 (78.0-85.0)	82.0 (78.0-86.0)	82.0 (78.0-85.0)	0.438	0.675	0.752
HOMA-IR	8.9 (6.4-12.3)	9.9 (7.2-14.2)	9.4 (6.6-13.5)	**<.001**	**0.042**	0.257
HOMA-B	879.7 (663.1-1218.5)	948.0 (665.1-1361.2)	928.3 (673.2-1344.6)	**0.038**	0.062	0.879
HbA1c (mmol/mol and %)	31.0 (29.0-32.0)	31.0 (29.0-33.0)	31.0 (29.0-32.0)	0.054	0.433	0.274
	5.0 (4.8-5.1)	5.0 (4.8-5.2)	5.0 (4.8-5.1)			
Fasting TG (mg/dl)	88.0 (70.0-109.5)	89.0 (73.0-114.0)	90.0 (71.0-117.0)	**0.032**	0.087	0.703
**24-28 weeks visit**						
BMI (Kg/m²)	26.4 ± 4.1	27.9 ± 5.2	27.9 ± 4.6	**<.001**	**<.001**	0.331
% Overweight	38.5 (310)	39.5 (182)	44.0 (213)	**<.001**	**<.001**	0.482
% Obesity	17.6 (142)	28.6 (132)	26.0 (126)			
Systolic blood pressure (mmHg)	112.7 ± 9.9	114.4 ± 10.6	113.5 ± 10.3	**0.014**	0.215	0.257
Diastolic blood pressure (mmHg)	67.0 ± 7.8	67.6 ± 8.3	67.5 ± 8.1	0.252	0.235	0.968
Total score lifestyle						
Physical activity	1.0 (0.0-2.0)	1.0 (0.0-2.0)	1.0 (0.0-2.0)	0.243	0.416	0.080
Diet	2.0 (0.0-4.0)	2.0 (0.0-4.0)	2.0 (-1.0-4.0)	0.260	0.059	0.540
IPAQ low	15.5 (126)	18.6 (84)	16.9 (80)	0.155	0.509	0.497
METs category:				0.095	0.791	0.335
% Low	15.5 (126)	18.6 (84)	16.9 (80)			
% Moderate	46.1 (375)	48.7 (220)	45.8 (217)			
% High	38.4 (313)	32.7 (148)	37.3 (177)			
% clinical depression	16.2 (135)	15.8 (74)	14.8 (73)	0.840	0.489	0.666
(≥16 on CES-D questionnaire)						
Glucose 60 min on GCT (mg/dl)	117.9 ± 27.8	121.0 ± 25.9	123.9 ± 27.7	**0.032**	**<.001**	0.139
Fasting glycaemia (mg/dl)	78.0 (74.0-82.0)	79.0 (75.0-83.0)	78.0 (74.0-83.0)	**0.005**	0.083	0.341
1-hour glucose OGTT (mg/dl)	126.0 (108.0-146.0)	128.5 (109.0-149.0)	131.0 (111.5-151.0)	0.191	**0.011**	0.311
2-hour glucose OGTT (mg/dl)	111.0 (95.0-129.0)	110.0 (95.0-130.0)	113.5 (95.0-132.0)	0.825	0.376	0.360
HbA1c	30.0 (29.0-32.0)	31.0 (29.0-32.0)	30.0 (29.0-32.0)	**<.001**	**0.031**	0.106
(mmol/mol and %)	4.9 (4.8-5.1)	5.0 (4.8-5.1)	4.9 (4.8-5.1)			
Matsuda insulin sensitivity	0.6 (0.4-0.8)	0.5 (0.4-0.7)	0.5 (0.4-0.8)	**0.006**	0.079	0.343
HOMA-IR	11.6 (8.5-16.9)	13.4 (9.5-18.8)	12.6 (9.0-17.9)	**0.009**	0.069	0.716
HOMA-B	1528.8 (1096.4-2259.0)	1588.1 (1139.3-2256.0)	1594.8 (1118.6-2248.0)	0.867	0.977	0.866
ISSI-2	0.1 (0.1-0.2)	0.1 (0.1-0.2)	0.1 (0.1-0.2)	0.061	0.528	0.265
Insulinogenic index/HOMA-IR	0.3 (0.2-0.5)	0.3 (0.2-0.5)	0.3 (0.2-0.4)	0.196	**0.003**	0.171
Fasting TG (mg/dl)	160.0 (128.0-202.0)	165.0 (133.0-206.0)	164.0 (132.0-207.0)	0.345	0.327	0.962
% Need for treatment with insulin (total)	1.0 (8)	3.4 (16)	2.2 (11)	**0.002**	0.092	0.330
% short acting insulin	0.2 (2)	0.9 (4)	0.4 (2)	**0.006**	0.179	0.616
% long acting insulin	0.6 (5)	1.1 (5)	1.0 (5)			
% short and long-acting insulin	0.1 (1)	1.5 (7)	0.8 (4)			
**Delivery**						
Total Weight gain (first visit till delivery) (Kg)	12.0 ± 4.5	11.2 ± 5.7	11.7 ± 5.3	**0.024**	0.242	0.408
% excessive weight gain	27.7 (205)	27.8 (113)	31.9 (138)	0.282	0.316	0.186
Gestational age (weeks)	39.3 ± 1.6	39.2 ± 1.5	39.2 ± 1.7	0.565	0.511	0.988
% Preeclampsia	1.2 (10)	2.3 (11)	2.0 (10)	0.114	0.238	0.722
% Gestational hypertension	3.4 (28)	5.3 (25)	4.6 (23)	0.083	0.242	0.615
% Preterm delivery	4.9 (41)	5.3 (25)	7.1 (35)	0.755	0.106	0.264
% Induction labor	26.6 (198)	32.5 (137)	33.2 (148)	**0.031**	**0.015**	0.841
% Forceps or vacuum	12.4 (103)	11.5 (54)	12.5 (62)	0.089	0.239	0.803
% Cesarean sections (total)	19.5 (162)	23.1 (108)	21.3 (105)	0.127	0.440	0.496
% Planned CS	11.3 (94)	10.0 (47)	9.7 (48)	0.089	0.239	0.803
% Emergency CS (during labor)	8.2 (68)	13.0 (61)	11.5 (57)	**0.005**	**0.043**	0.480
Weight baby (g)	3391.7 ± 498.1	3393.0 ± 482.4	3375.2 ± 541.7	0.908	0.675	0.827
% Macrosomia (>4Kg)	9.3 (77)	8.8 (41)	9.1 (45)	0.746	0.917	0.842
% Weight baby ≥4.5Kg	1.4 (9)	1.1 (5)	1.4 (7)	0.979	0.592	0.626
% LGA	12.9 (107)	12.6 (59)	12.1 (60)	0.894	0.693	0.818
% SGA	5.2 (43)	4.9 (23)	4.5 (22)	0.844	0.556	0.729
% Apgar 10min <7	1.3 (11)	0.0 (0)	1.0 (5)	**0.010**	0.796	0.062
%Shoulder dystocia	0.8 (7)	1.1 (5)	1.2 (6)	0.685	0.508	0.831
% Congenital anomaly	4.2 (35)	4.5 (21)	3.8 (19)	0.887	0.776	0.632
% Respiratory Distress syndrome	1.2 (10)	0.4 (2)	1.0 (5)	0.230	0.796	0.453
% Neonatal hypoglycemia <40mg/dl	4.6 (26)	5.6 (17)	7.8 (26)	0.543	0.050	0.261
% Neonatal jaundice	17.5 (100)	21.0 (72)	18.8 (68)	0.186	0.609	0.462
% NICU admission	10.0 (83)	9.2 (43)	10.9 (54)	0.616	0.602	0.364
Days on NICU	8.1 ± 13.4	8.4 ± 13.3	8.6 ± 13.7	0.757	0.538	0.442
**Postpartum**						
% Postpartum OGTT	9.0 (92)	12.5 (59)	12.5 (62)	**0.045**	**0.043**	0.991
% glucose intolerance				0.122	0.082	0.084
None	80.0 (60)	88.1 (52)	80.6 (50)			
IFG	10.7 (8)	5.1 (3)	1.6 (1)			
IGT	9.3 (7)	3.4 (2)	16.1 (10)			
IFG+IGT	0.0 (0)	3.4 (2)	1.61 (1)			
% breastfeeding	85.5 (65)	81.0 (47)	80.0 (48)	0.487	0.393	0.887
Lifestyle score:						
Physical activity	1.0 (0.0-1.0)	1.0 (0.0-2.0)	1.0 (0.0-2.0)	0.432	0.170	0.379
Diet	5.0 (1.0-7.0)	2.0 (-1.0-4.0)	2.0 (0.0-5.0)	**0.048**	0.202	0.366
Energy	62.5 (50.0-75.0)	62.5 (50.0-75.0)	62.5 (50.0-75.0)	0.652	0.883	0.776
Emotional Wellbeing	70.0 (65.0-75.0)	70.0 (65.075.0)	70.0 (65.0-75.0)	0.793	0.326	0.519
Social functioning	87.5	87.5	87.5	0.697	0.842	0.866
	(75.0-100.0)	(75.0-100.0)	(75.0-100.0)			
Pain	90.0 (77.5-100.0)	90.0 (77.5-100.0)	90.0 (77.5-100.0)	0.750	0.482	0.390
General Health	75.0 (65.0-85.0)	75.0 (65.0-85.0)	75.0 (60.0-85.0)	**0.046**	0.217	0.583
Health Transition	50.0 (50.0-50.0)	50.0 (50.0-50.0)	50.0 (50.0-50.0)	0.513	0.882	0.626

OGTT, oral glucose tolerance test; GDM, gestational diabetes mellitus; BMI, Body Mass Index; HDL, high-density lipoprotein; LDL, low-density-lipoprotein; TG, triglycerides; MET, metabolic equivalent of task; LGA, large-for-gestational age infant; SGA, small-for-gestational age infant; NICU, neonatal intensive care unit; IFG, impaired fasting glycemia; IGT, impaired glucose tolerance; SF-36, 36-Item Short Form Health Survey; CES-D, Center for Epidemiologic Studies – Depression. Overweight: BMI ≥25-29.9 Kg/m²; Obesity: BMI ≥30 Kg/m². Questionnaires in the postpartum period were only administered by women with GDM who attended the OGTT. Categorical variables are presented as frequencies %(n); continuous variables are presented as mean ± SD if normally distributed and as median ± IQR if not normally distributed; Differences are considered significant at p-value<0.05. *A history of GDM and a history of a macrosomic baby were calculated on the number of women with a previous pregnancy. Bold value means that this is significant, meaning that the p-value < 0.05.

### Characteristics of Women According to the Preference of Screening Test

Compared to women who preferred an OGTT, women who preferred a GCT had less often a minor ethnic background, had less often a previous history of GDM, had a lower BMI [24.1 ± 4.2 *vs*. 25.7 ± 6.1, p=0.023], and were less often overweight or obese [respectively 22.3% (219) *vs*. 25.0% (28), p=0.005 and 10.1% (99) *vs*. 19.6% (22), p=0.005]. At 24-28 weeks of pregnancy, significantly more women needed treatment with insulin for GDM in the group who preferred an OGTT compared to the group who preferred a GCT [5.4% (6) *vs*. 1.1% (11), p=0.005] (**Table 4**). There was no difference in the multiparity rate between both groups.

**Table 4:** Comparison of characteristics and pregnancy outcomes between women who prefer a GCT compared to women who prefer an OGTT or without clear preference.

	Preference OGTT	Preference GCT	No preference	Pairwise comparisons		
	N = 112 (6.21%)	N = 989 (54.82%)	N = 703 (38.97%)			
				1 *vs* 2	1 *vs* 3	2 *vs* 3
**General**						
Mean age (years)	31.0 ± 3.8	30.7 ± 4.0	30.8 ± 4.1	0.464	0.587	0.600
% Minor ethnicities	12.6 (14)	6.8 (67)	11.3 (79)	**0.027**	0.691	**0.001**
% multiparity	51.8 (58)	47.1 (466)	47.2 (332)	0.349	0.370	0.965
% Highest education:				0.099	0.396	**<.001**
primary school	1.8 (2)	0.6 (6)	1.7 (12)			
till 15 years	3.6 (4)	2.8 (27)	6.6 (45)			
high school	20.0 (22)	14.7 (144)	20.4 (140)			
bachelor	47.3 (52)	43.2 (423)	38.5 (264)			
master	27.3 (30)	38.6 (378)	32.8 (225)			
% paid job	91.0 (101)	93.0 (915)	90.6 (634)	0.441	0.888	0.072
% low monthly net income family <1500 euro	3.6 (4)	2.4 (23)	6.1 (42)	0.547	0.516	**<.001**
% 1500-5000 euro	91.0 (101)	90.6 (876)	89.6 (614)			
% >5000 euro	5.4 (6)	7.0 (68)	4.2 (29)			
Low income (<1500 euro)				0.514	0.382	**<.001**
% No	96.4 (107)	97.6 (944)	93.9 (643)			
% Yes	3.6 (4)	2.4 (23)	6.1 (42)			
% living without partner	15.2 (17)	15.9 (156)	20.6 (144)	0.849	0.184	**0.013**
% smoking before pregnancy	25.9 (29)	31.3 (309)	27.4 (191)	0.237	0.739	0.082
% smoking during pregnancy	2.7 (3)	4.0 (40)	3.1 (22)	0.613	1.000	0.360
% First degree family history of diabetes	13.8 (15)	11.3 (108)	14.5 (100)	0.448	0.844	0.057
% First degree family history of GDM	5.7 (6)	4.1 (37)	4.5 (30)	0.443	0.617	0.645
% History of GDM*	21.4 (12)	8.2 (39)	7.1 (24)	**0.002**	**<.001**	0.562
% history of PCOS	11.6 (13)	6.9 (68)	7.0 (49)	0.071	0.088	0.934
**6-14 weeks visit**						
Week first visit with FPG	11.9 ± 1.6	11.8 ± 1.8	12.0 ± 1.7	0.269	0.906	**0.041**
BMI (Kg/m²)	25.7 ± 6.1	24.1 ± 4.2	25.4 ± 4.8	**0.023**	0.752	**<.001**
% Overweight	25.0 (28)	22.3 (219)	29.4 (205)	**0.005**	0.680	**<.001**
% Obesity	19.6 (22)	10,1 (99)	15.2 (106)			
Waist circumference (cm)	88.4 ± 14.4	86.0 ± 10.4	88.4 ± 11.7	0.166	0.741	**<.001**
% Waist ≥80cm	74.3 (81)	74.0 (702)	76.9 (515)	0.155	0.837	**<.001**
Weight gain (first visit till OGTT) (Kg)	7.0 ± 4.0	7.1 ± 3.0	7.0 ± 3.6	0.237	0.251	0.884
Systolic blood pressure (mmHg)	115.6 ± 10.6	114.6 ± 10.7	115.5 ± 10.5	0.315	0.929	**0.049**
Diastolic blood pressure (mmHg)	71.3 ± 8.4	70.2 ± 8.0	70.9 ± 8.4	0.280	0.695	0.149
Total Score lifestyle						
Physical activity	1.0 (0.0-2.0)	1.0 (0.0-2.0)	1.0 (0.0-2.0)	0.069	0.167	0.487
Diet	2.0 (1.0-5.0)	2.0 (0.0-4.0)	1.0 (0.0-4.0)	0.050	**0.007**	0.137
Fasting glycaemia (mg/dl)	83.0 (78.0-86.0)	81.0 (78.0-85.0)	82.0 (78.0-86.0)	0.146	0.436	0.191
Fasting insulin (pmol/l)	50.1 (34.0-70.7)	44.6 (33.0-61.3)	47.5 (34.4-68.2)	**0.043**	0.515	**0.006**
HOMA-IR	10.1 (6.9-14.4)	8.9 (6.5-12.5)	9.6 (6.7-14.2)	**0.038**	0.526	**0.004**
HOMA-B	953.4 (715.1-1307.4)	890.3 (648.0-1239.8)	936.0 (687.0-1353.6)	0.210	0.946	**0.020**
HbA1c (mmol/mol and %)	31.0 (29.0-33.0)	31.0 (29.0-32.0)	31.0 (29.0-33.0)	0.272	0.763	0.082
	5.0 (4.8-5.2)	5.0 (4.8-5.1)	5.0 (4.8-5.2)			
Fasting TG (mg/dl)	89.0 (74.0-118.0)	89.0 (71.0-111.0)	89.0 (71.0-114.0)	0.187	0.297	0.597
**24-28 weeks visit**						
BMI (Kg/m²)	28.3 ± 6.0	26.6 ± 4.2	27.8 ± 4.7	**0.007**	0.726	**<.001**
% Overweight	33.6 (37)	38.8 (372)	42.9 (293)	**<.001**	**0.001**	**<.001**
% Obesity	34.5 (38)	19.5 (187)	25.8 (176)			
Systolic blood pressure (mmHg)	115.0 ± 12.7	112.9 ± 9.8	113.7 ± 10.4	0.174	0.431	0.217
Diastolic blood pressure (mmHg)	67.6 ± 9.4	67.2 ± 7.8	67.4 ± 8.0	0.822	0.931	0.487
Total score lifestyle						
Physical activity	1.0(0.0-2.0)	1.0(0.0-2.0)	1.0(0.0-2.0)	0.322	0.717	0.212
Diet	3.0(1.0-5.0)	2.0(0.0-4.0)	2.0(-1.0-4.0)	**0.033**	**0.003**	0.056
IPAQ low	17.0 (18)	16.2 (156)	17.3 (116)	0.833	0.938	0.555
METs category:				0.975	0.737	0.284
% Low	17.0 (18)	16.2 (156)	17.3 (116)			
% Moderate	48.1 (51)	48.2 (465)	44.3 (297)			
% High	34.9 (37)	35.6 (343)	38.4 (258)			
% clinical depression	11.6 (13)	16.8 (166)	14.7 (103)	0.156	0.380	0.246
(≥16 on CES-D questionnaire)						
Glucose 60 min on GCT (mg/dl)	122.6 ± 24.5	119.0 ± 27.1	122.2 ± 28.4	0.123	0.613	0.059
Fasting glycaemia (mg/dl)	79.0 (75.0-85.0)	78.0 (74.0-82.0)	79.0 (75.0-83.0)	**0.042**	0.448	**0.006**
1-hour glucose OGTT (mg/dl)	132.0 (110.0-154.0)	127.0 (108.0-146.0)	129.0 (110.0-150.0)	0.211	0.640	0.108
2-hour glucose OGTT (mg/dl)	116.0 (98.0-136.0)	111.5 (94.0-129.5)	111.0 (95.0-130.0)	0.125	0.115	0.843
HbA1c	31.0 (29.0-33.0)	30.0 (29.0-32.0)	30.0 (29.0-32.0)	0.066	0.446	**0.019**
(mmol/mol and %)	5.0 (4.8-5.1)	4.9 (4.8-5.1)	4.9 (4.8-5.1)			
Matsuda insulin sensitivity	0.5(0.3-0.8)	0.6 (0.4-0.8)	0.5(0.4-0.7)	0.803	0.410	0.159
HOMA-IR	13.7 (9.0-21.1)	11.8 (8.6-16.8)	13.0 (9.1-18.1)	0.140	0.772	0.050
HOMA-B	1728.4 (1107.8-2268.0)	1548.0 (1123.7-2259.0)	1568.6 (1122.9-2241.0)	0.723	0.627	0.888
ISSI-2	0.1 (0.1-0.2)	0.1 (0.1-0.2)	0.1 (0.1-0.2)	0.338	0.137	0.188
Insulinogenic index/HOMA-IR	0.3 (0.2-0.4)	0.3 (0.2-0.5)	0.3 (0.2-0.4)	0.487	0.897	0.255
Fasting Total cholesterol (mg/dl)	248.0 (221.5-270.0)	243.0 (220.0-273.0)	241.0 (216.0-273.0)	0.901	0.529	0.121
Fasting HDL (mg/dl)	75.0 (64.5-85.5)	74.0 (64.0-87.0)	74.0 (64.0-86.0)	0.835	0.957	0.704
Fasting LDL (mg/dl)	135.0 (112.0-153.0)	134.0 (113.0-161.0)	131.0 (109.0-159.0)	0.551	0.740	0.059
Fasting TG (mg/dl)	165.0 (141.0-216.5)	160.0 (127.0-204.0)	165.0 (133.0-202.0)	0.057	0.161	0.276
Increase (difference) in TG between first and second visit (mg/dl)	74.0 (52.0-112.0)	70.0 (43.0-101.0)	71.0 (47.0-99.0)	0.143	0.239	0.462
% Need for treatment with insulin (total)	5.4 (6)	1.1 (11)	2.6 (18)	**0.005**	0.125	**0.035**
% short acting insulin	0.9 (1)	0.6 (6)	1.1 (8)	**<.001**	0.153	0.088
% long acting insulin	1.8 (2)	0.3 (3)	0.4 (3)			
% short and long-acting insulin	2.7 (3)	0.2 (2)	1.0 (7)			
**Delivery**						
Total Weight gain (first visit till delivery) (Kg)	11.7 ± 4.3	11.9 ± 4.6	11.3 ± 5.8	0.496	0.675	**0.026**
% excessive weight gain	30.4 (28)	27.6 (241)	30.7 (188)	0.840	0.942	0.251
Gestational age (weeks)	39.1 ± 1.7	39.3 ± 1.6	39.2 ± 1.6	0.626	0.967	0.273
% Preeclampsia	0.9 (1)	1.4 (14)	2.3 (16)	1.000	0.716	0.196
% Gestational hypertension	4.5 (5)	3.3 (33)	5.4 (38)	0.515	0.706	**0.037**
% Preterm delivery	6.3 (7)	5.2 (51)	6.2 (43)	0.618	0.953	0.393
% Induction labor	26.0 (26)	27.7 (246)	33.9 (212)	0.713	0.120	**0.011**
% Forceps or vacuum	14.4 (16)	11.9 (117)	12.2 (85)	0.167	0.532	0.444
% Cesarean sections (total)	25.2 (28)	19.9 (196)	21.5 (150)	0.167	0.532	0.444
% Planned CS	9.9 (11)	10.6 (104)	10.6 (74)	0.167	0.532	0.444
% Emergency CS (during labor)	15.3 (17)	9.3 (92)	10.9 (76)	**0.046**	0.174	0.297
% Postpartum blood loss				0.655	0.847	0.798
≥500ml	21.1 (23)	20.9 (204)	20.4 (141)			
≥1000ml	3.7 (4)	2.3 (22)	2.7 (19)			
Weight baby (g)	3358.5 ± 510.9	3394.2 ± 509.5	3382.7 ± 503.0	0.460	0.619	0.575
% Macrosomia (>4Kg)	9.0 (10)	9.5 (93)	8.6 (60)	0.877	0.893	0.556
% Weight baby ≥4.5Kg	0.9 (1)	1.4 (14)	0.9 (6)	1.000	1.000	0.365
% LGA	11.7 (13)	13.2 (130)	12.0 (84)	0.654	0.923	0.471
% SGA	4.5 (5)	4.8 (47)	5.3 (37)	0.895	0.721	0.624
% Apgar 10min <7	0.0 (0)	1.1 (11)	0.7 (5)	0.615	1.000	0.457
%Shoulder dystocia	0.0 (0)	1.5 (15)	0.4 (3)	0.387	1.000	**0.032**
% Congenital anomaly	4.5 (5)	4.4 (43)	3.7 (26)	1.000	0.603	0.535
% Respiratory Distress syndrome	0.0 (0)	1.1 (11)	0.9 (6)	0.616	1.000	0.632
% Neonatal hypoglycemia <40mg/dl	2.8 (2)	5.8 (39)	6.1 (28)	0.416	0.407	0.799
% Neonatal jaundice	17.9 (15)	18.9 (129)	18.9 (96)	0.820	0.815	0.983
% NICU admission	16.2 (18)	9.3 (91)	10.4 (72)	**0.021**	0.069	0.457
Days on NICU	7.2 ± 9.5	7.9 ± 13.1	8.9 ± 14.7	0.817	0.671	0.679
**Postpartum**						
% Postpartum OGTT	15.2 (17)	9.2 (91)	12.5 (88)	**0.044**	0.435	**0.029**
% glucose intolerance				0.416	0.514	0.437
IFG	5.9 (1)	8.8 (8)	3.4 (3)			
IGT	0.0 (0)	11.0 (10)	10.2 (9)			
IFG+IGT	0.0 (0)	2.2 (2)	1.1 (1)			
% breastfeeding	81.2 (13)	84.6 (77)	80.5 (70)	0.734	0.941	0.465
Lifestyle score:						
Physical activity	1.0 (0.0-1.0)	1.0 (0.0-2.0)	1.0 (0.0-2.0)	0.432	0.170	0.379
Diet	5.0 (1.0-7.0)	2.0 (-1.0-4.0)	2.0 (0.0-5.0)	**0.048**	0.202	0.366
SF36						
Physical functioning	85.7 (80.0-95.0)	90.0 (83.3-100.0)	90.0 (80.0-100.0)	0.062	0.089	0.982
Role physical	81.2 (62.5-100.0)	87.5 (65.6-100.0)	87.5 (68.7-100.0)	0.806	0.999	0.566
Role Emotional	100.0 (66.7-100.0)	100.0 (75.0-100.0)	100.0 (66.7-100.0)	0.685	0.760	0.854
Energy	62.5 (56.2-75.0)	62.5 (50.0-75.0)	62.5 (50.0-75.0)	0.078	0.419	0.056
Emotional Wellbeing	70.0 (65.0-75.0)	70.0 (65.0-75.0)	70.0 (65.0-75.0)	0.218	0.255	0.903
Social functioning	100.0 (75.0-100.0)	87.5 (75.0-100.0)	87.5 (75.0-100.0)	0.335	0.845	0.109
Pain	80.0 (67.5-100.0)	90.0 (77.5-100.0)	90.0 (77.5-100.0)	0.093	**0.028**	0.330
General Health	75.0 (65.0-85.0)	75.0 (65.0-85.0)	75.0 (65.0-85.0)	0.640	0.857	0.641
Health Transition	50.0 (50.0-50.0)	50.0 (50.0-50.0)	50.0 (50.0-50.0)	0.541	0.925	0.266
METs category:				1.000	0.489	0.065
% Low	13.3 (2)	17.0 (15)	7.6 (6)			
% Moderate	53.3 (8)	51.1 (45)	45.6 (36)			
% High	33.3 (5)	31.8 (28)	46.8 (37)			
% clinical depression	11.8 (2)	17.4 (16)	15.9 (14)	0.566	0.663	0.790
(≥16 on CES-D questionnaire)						

OGTT, oral glucose tolerance test; GCT, glucose challenge test; GDM, gestational diabetes mellitus; BMI, Body Mass Index; HDL, high-density lipoprotein; LDL, low-density-lipoprotein; TG, triglycerides; MET, metabolic equivalent of task; LGA, large-for-gestational age infant; SGA, small-for-gestational age infant; NICU, neonatal intensive care unit; IFG, impaired fasting glycemia; IGT, impaired glucose tolerance; SF-36, 36-Item Short Form Health Survey; CES-D, Center for Epidemiologic Studies – Depression. Overweight: BMI ≥25-29.9 Kg/m²; Obesity: BMI ≥30 Kg/m². Questionnaires in the postpartum period were only administered by women with GDM who attended the OGTT. Categorical variables are presented as frequencies %(n); continuous variables are presented as mean ± SD if normally distributed and as median ± IQR if not normally distributed; Differences are considered significant at p-value<0.05. *A history of GDM and a history of a macrosomic baby were calculated on the number of women with a previous pregnancy. Bold value means that this is significant, meaning that the p-value < 0.05.

### Pregnancy Outcomes

Pregnancy outcomes were similar between women who preferred a one-step or two-step screening strategy, except for a lower rate of labor inductions and emergency cesarean sections (CS) in the group who preferred a two-step screening [respectively 26.6% (198) *vs*. 32.5% (137), p=0.031 and 8.2% (68) *vs*. 13.0% (61), p=0.005] (**Table 3**).

Women who preferred a GCT had less often emergency CS and less often neonatal jaundice [respectively: 9.3% (92) *vs*. 15.3% (92), p=0.046 and 9.3% (91) *vs*. 16.2% (18), p=0.021] compared to women who preferred an OGTT (**Table 4**).

### Postpartum Outcomes

Women who preferred an OGTT had a better diet score postpartum compared to women who preferred a GCT. There was no difference in rate of glucose intolerance postpartum between the different groups (**Tables 3**, **4**).

## Discussion

We found that the majority of pregnant women preferred a two-step screening strategy with a GCT for GDM. In addition, we show that the preference of GDM screening method differed by metabolic risk profile of participants and tolerance for the screening tests. Women with a more adverse metabolic profile preferred a one-step screening approach with OGTT while women preferring a two-step screening strategy had a better metabolic profile and more discomfort of the OGTT.

Several international societies such as the IADPSG and WHO recommend a one-step screening approach for GDM with a 75g OGTT (8, 9). However, this leads to an important increase in the number of women diagnosed with GDM and important increase in workload. Moreover, this could also lead to increased medicalization of care with more labor inductions and CS. Evidence is lacking that treatment of GDM based on the one-step IADPSG screening approach improves pregnancy outcomes compared to other screening strategies. Recently, two large RCT’s from the US showed that a one-step screening strategy with the IADPSG criteria leads to a 2-3 fold increase in GDM prevalence compared to screening with a two-step approach with GCT but without improvement of pregnancy outcomes (11). In addition, the OGTT is often considered a cumbersome test during pregnancy. In our study, nearly half of all women indicated that it was difficult to come fasting. When choosing a GDM screening approach, it is therefore also important to take into account the preference of pregnant women for the GDM screening method and tolerance of the screening tests. To our knowledge, our cohort is the first study to systematically assess the preference of pregnant women for GDM screening method and tests. Our results show that nearly half of all women preferred a two-step screening strategy over a one-step screening approach with OGTT. Women who preferred a two-step screening strategy tolerated the GCT in general better than the OGTT compared to women who preferred a one-step screening approach or women without clear preference. More women preferred therefore a GCT as screening test. This is in line with other studies reporting difficulties with an OGTT in pregnancy, in which vomiting is often a reason for failure of the test (30). A recent RCT from the US showed that a 75g OGTT was better tolerated than a 100g OGTT for the diagnosis of GDM (11). However, when using a two-step screening strategy with GCT, a 100g OGTT is only needed in about 20% of all pregnant women. In line with normal clinical practice, adverse events of the screening tests would therefore occur in only 4% of women using a two-step screening strategy with GCT compared to 13% in women undergoing the one-step IADPSG approach with OGTT (11).

Women who preferred a GCT or two-step screening strategy had a better metabolic profile (were less often obese and less insulin resistant) and had less risk factors for GDM compared to women who preferred an OGTT or one-step screening approach. We have previously demonstrated that women with a higher risk-profile, such as women with a previous history of GDM and higher BMI have the highest risk to develop GDM and would therefore benefit from a one-step approach with OGTT (10). The Flemish consensus on screening for GDM was revised in 2019 based on the BEDIP-N study. A modified two-step screening strategy for GDM with GCT and also based on risk-factors, was proposed to limit the number of missed cases with GDM and at the same time avoid an OGTT in about 50% of all pregnant women (16). Based on this modified two-step screening strategy, women at higher risk for GDM (women with a history of GDM, obesity and/or impaired fasting glycaemia in early pregnancy), are recommended a one-step screening strategy with an OGTT, while women without these risk factors, are offered a two-step screening strategy with GCT (13). With current study we show now that this screening approach also fits with the preference of women for GDM screening method according to their metabolic risk profile and tolerance of the tests. In our study, the preference of GDM screening method was not different in women who had been pregnant before and had already experienced screening for GDM. However, most women with a previous history of GDM preferred a one-step screening strategy with OGTT. This is probably due to the fact that these women perceive themselves to be at high risk for a recurrent diagnosis of GDM and will therefore more often need an OGTT (irrespective of screening approach).

Pregnancy outcomes were in general similar irrespective of the preference of the GDM screening method, expect for lower rates of labor inductions and emergency CS in women who preferred a two-step screening strategy. This is probably due to the lower metabolic risk of women who preferred two-step screening. In addition, research has shown that a higher income and a higher education leads to less inductions and emergency CS (31, 32). There was no difference in the rate of glucose intolerance postpartum between both groups. Women who preferred an OGTT had a higher diet score, suggesting a healthier diet in early postpartum. This might be due to the fact that they perceive themselves at higher risk to develop diabetes postpartum.

Strengths of our study are the large prospective cohort with detailed data on clinical characteristics and obstetrical outcomes. In addition, women were blinded for the result of the GCT, so that they could not be biased by this result and their preference for a GCT or OGTT. Moreover, the tolerance and preference of GDM screening method was systematically recorded at the time of the GCT and OGTT. A limitation of the study is that the cohort consisted mostly of a Caucasian population with a rather low background risk for GDM. In addition, we did not perform extensive interviews to assess the tolerance of tests and reasons for the preference of GDM screening method.

In conclusion, our study showed that most women preferred a two-step screening strategy with GCT for GDM. In addition, we show that the preference of GDM screening method differed by metabolic risk profile of participants and tolerance of tests.

## Data Availability Statement

The raw data supporting the conclusions of this article will be made available by the authors, without undue reservation.

## Ethics Statement

The studies involving human participants were reviewed and approved by Ethics committee of UZ Leuven, Leuven, Belgium. The patients/participants provided their written informed consent to participate in this study.

## Author Contributions

KB, PC, and CMa conceived the project. CMo prepared the data and ALa did the statistical analysis. LR did the literature review. LR and KB wrote the first draft of the manuscript. All authors contributed to the study design, including data collection, data interpretation and manuscript revision. The corresponding author LR had full access to all the data in the study and had final responsibility for the contents of the article and the decision to submit for publication. All authors contributed to the article and approved the submitted version.

## Funding

This investigator-initiated study was funded by the Belgian National Lottery, the Fund of the Academic studies of UZ Leuven, and the Fund Yvonne and Jacques François-de Meurs of the King Boudewijn Foundation. The funders were not involved in the study design, collection, analysis, interpretation of data, the writing of this article or the decision to submit it for publication.

## Conflict of Interest

The authors declare that the research was conducted in the absence of any commercial or financial relationships that could be construed as a potential conflict of interest.

## Publisher’s Note

All claims expressed in this article are solely those of the authors and do not necessarily represent those of their affiliated organizations, or those of the publisher, the editors and the reviewers. Any product that may be evaluated in this article, or claim that may be made by its manufacturer, is not guaranteed or endorsed by the publisher.
