# Supplementary material for: Preference of Women for Gestational Diabetes Screening Method According to Tolerance of Tests and Population Characteristics
Source: Front Endocrinol (Lausanne). 2021 Nov 8;12:781384. doi: 10.3389/fendo.2021.781384 (PMC8630544; doi:10.3389/fendo.2021.781384)
Supplement: Supplementary file 1 [file DataSheet_1.docx]

Supplementary Material

# Questionnaire on the evaluation of the discomfort of the glucose challenge test:

1.Did you experience any discomfort with the test? □yes □no

2.If you had any discomfort, which complaint or complaints did you have with this test (multiple answers are possible)

□bad taste

□nausea

□vomiting

□dizziness or feeling faint

□abdominal pain

□other: which?: …

# Questionnaire on the evaluation of the discomfort of the OGTT:

1.Did you experience any discomfort with the test? □yes □no

2.If you had any discomfort, which complaint or complaints did you have with this test (multiple answers are possible)

□bad taste

□nausea

□vomiting

□dizziness or feeling faint

□abdominal pain

□other: which?: …

3.Do you find it cumbersome that you have to be fasting fort his test?

□yes □no

4. If you could choose which sreening test would be used, would you prefer the non-fasting 50g glucose test above the fasting glucose tolerance test?

□yes □no □no preference

5. Which screening test would you prefer:

□a non-fasting 50g glucose test and only when abnormal, followed by a fasting glucose tolerance test

□immediately a fasting glucose tolerance test so that you would certainly only need one test

□no preference
